# Distinguishing mirtrons from canonical miRNAs with data exploration and machine learning methods

**DOI:** 10.1038/s41598-018-25578-3

**Published:** 2018-05-15

**Authors:** Grzegorz Rorbach, Olgierd Unold, Bogumil M. Konopka

**Affiliations:** 1 Department of Computer Engineering, Faculty of Electronics, Wroclaw University of Science and Technology, Wroclaw, Poland; 2 Department of Biomedical Engineering, Faculty of Fundamental Problems of Technology, Wroclaw University of Science and Technology, Wroclaw, Poland

## Abstract

Mirtrons are non-canonical microRNAs encoded in introns the biogenesis of which starts with splicing. They are not processed by Drosha and enter the canonical pathway at the Exportin-5 level. Mirtrons are much less evolutionary conserved than canonical miRNAs. Due to the differences, canonical miRNA predictors are not applicable to mirtron prediction. Identification of differences is important for designing mirtron prediction algorithms and may help to improve the understanding of mirtron functioning. So far, only simple, single-feature comparisons were reported. These are insensitive to complex feature relations. We quantified miRNAs with 25 features and showed that it is impossible to distinguish the two miRNA species using simple thresholds on any single feature. However, when using the Principal Component Analysis mirtrons and canonical miRNAs are grouped separately. Moreover, several methodologically diverse machine learning classifiers delivered high classification performance. Using feature selection algorithms we found features (e.g. bulges in the stem region), previously reported divergent in two classes, that did not contribute to improving classification accuracy, which suggests that they are not biologically meaningful. Finally, we proposed a combination of the most important features (including Guanine content, hairpin free energy and hairpin length) which convey a specific pattern, crucial for identifying mirtrons.

## Introduction

MicroRNAs (miRNAs) are a class of short (≈22 nt), non-coding RNA molecules^1^. They regulate gene expression at the post-transcriptional level^2^. Their canonical biogenesis pathway starts with transcription from independent genes, which forms primary miRNA hairpins (pri-miRNA)^3^. This is followed by cleavage performed by the Microprocessor complex, consisting of Drosha and DGCR8 proteins^4^, which produces a stem-loop precursor miRNA referred to as pre-miRNA hairpin. Pre-miRNA is then transported to the cytosol by exportin-5^5^ and is further processed by the enzyme Dicer. The enzyme cleaves the terminal loop, leaving a miRNA duplex. Generally it is assumed that only one strand of the duplex is functional and joins the Argonaute protein to form the RNA-induced silencing complex (RISC)^6^, while the other strand is degraded. However, recent short-read NGS data show that many hairpins produce functional mature miRNA from both duplex arms^7^. Functional, mature miRNA guides RISC to the target mRNAs through complementary binding, which leads to suppression of translation or accelerated degradation^2^. A multitude of studies have shown that miRNAs may be aberrantly expressed in various states, e.g. in cancer^8–10^, vascular diseases^11,12^ or inflammation^13–16^. Recently efforts are made to use specific miRNAs as diagnostic or therapeutic agents^17,18^. Also there is evidence that miRNAs participate in host-microbiome communication^19^.

Mirtrons are miRNAs originating from a non-canonical biogenesis pathway that omits Drosha cleavage^20^. They are byproducts of intron splicing. Mirtrons were first discovered as short introns that formed hairpins with similar characteristics to those of pre-miRNAs^21,22^, i.e. conserved stem regions and variable terminal loop^21^. Those pre-miRNAs undergo lariat-debranching by a debranching enzyme (DBR1) and enter the canonical miRNA biogenesis pathway at the exportin-5 level. These are often called canonical mirtrons. There are also two other types of mirtrons called 3′-tailed and 5′-tailed mirtrons^20^. These molecules undergo lariat-debranching by the DRB1 protein and 5′ or 3′ trimming by RNA exosome. Afterwards they similarly enter the canonical miRNA biogenesis pathway at the exportin-5 stage. Although the conservation patterns of mirtrons and canonical miRNAs are similar, only few mirtrons are evolutionarily conserved. For instance in a study by Wen *et al*.^23^ it was shown that human and mouse genomes share only 13 mirtrons out of a total of 478 and 488 mirtrons reported respectively.

Mirtrons were characterized in multiple experimental studies carried out on invertebrate^21,22,24^, mammalian^23,25^ and plant samples. Most recent studies were based on the analysis of small RNA NGS datasets. These works reported on the differences between canonical and non-canonical miRNAs and tried to determine specific mirtron structural characteristics and sequence patterns. It was shown that all mirtron types in comparison to bulk intronic sequences, exhibit higher GC content in the duplex regions, which also results in lower free energy (FE)^21,23,25^. In comparison to canonical miRNAs mirtron hairpins are in general longer and show a higher rate of internal loops and bulges^26,27^. Another important structural feature is the overhang, i.e. a short unpaired sequence of nucleotides on the stem end of the molecule. Canonical pre-miRNAs exhibit a typical 0:2 (5′:3′) AG overhang as a result of Drosha cleavage. The overhang was reported as optimal for recognition by exportin-5^26^. Mirtrons that are derived directly from splicing (canonical mirtrons) have usually a 1:1 nucleotide overhang with a G from the GU splice donor at the 5′ end and a G from the AG splice acceptor on the 3′ end^21^. However, for other mirtron types other configurations are also possible, e.g. 2:3, 0:3^26^. The most common sequence patterns of mirtrons come from the fact that they are partly produced by the precise splicing machinery. Thus, the exon-neighboring mirtron ends are dominated by GU’s in case of 5p arms of canonical and 3′-tailed mirtrons, and AG’s in case of 3p arms of canonical and 5′-tailed mirtrons^26,27^. Moreover the 3p arms of 5-tailed mirtrons are pyrimidine rich due to the polypirimidine tract within intron^23,24^. In case of some 3′ tailed mirtrons the GU in 5p arms may be substituted with xU due to the action of a 5-directed exoribonuclease^27^.The above characteristics are simple and based on single features and may miss more complex relations and dependencies. In the presented work we use more advanced computational tools to investigate the canonical miRNA vs mirtron differences in a multidimensional space.

**Figure 1:**
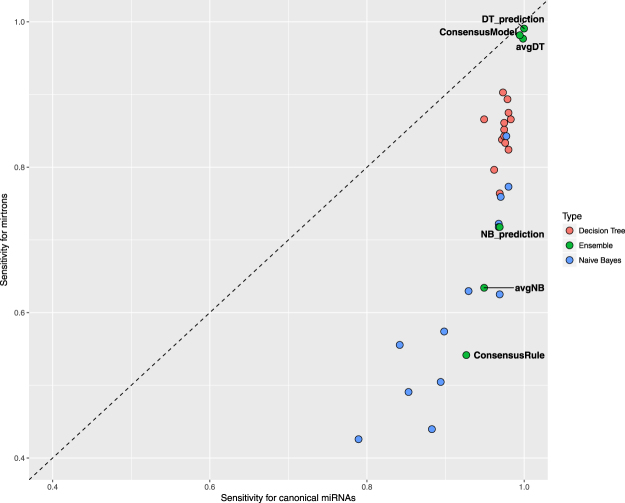
Performance of state-of-the-art miRNA predictors is worse in case of mirtrons than in case of canonical miRNAs. The dashed line denotes equal performance, points above the line denote higher performance for mirtrons, points below denote higher performance for canonical miRNAs. The sensitivities delivered by miRNA predictors available through izMIR framework^40^ (Decision Tree-based - red, Naive Bayes-based - blue, Ensemble - green) were always higher for canonical miRNAs (below dashed line). For the sake of clarity only labels of ensemble predictors were printed.

There are many tools for computational prediction of miRNAs which are based on diverse methodologies. So far among the most successful were methods based on SVM^28–34^. However, other approaches were also tested, e.g. Random Forest classifier was used in MiPred^35^ and was also chosen as best performing method in HuntMi^36^. A novel Markov random walk based method was implemented in miRank^37^, while deKmer^38^ is a quantum mechanics inspired method. Usually, new tools are developed with the use of enlarged feature sets and new, larger or improved data sets. Several studies emphasized on the influence of the training set class balance and the negative sample set composition on predictor performance^34,36,39^. In general, each new study shows that the new tool outperforms all remaining ones. However due to the differences in training and test sets a reliable comparison of performance is difficult. Only recently Saçar Demirci *et al*. developed a framework - izMiR^40^ and performed a large scale comparison of 13 state-of-art miRNA predictors. They concluded that consensus predictors provide the highest performance but none of the single predictors reliably outperforms the others. Our re-analysis of data provided by Saçar Demirci *et al*.^40^ showed that most predictors acquired considerably lower sensitivity for mirtrons than for canonical miRNAs (Fig. 1 and Supplementary Table S1). This shows that dividing the problem of miRNA prediction into canonical and non-canonical miRNA prediction may lead to further improvement of the field.

So far there were only a few attempts to develop computational models dedicated to mirtron prediction. Chung *et al*.^26^ developed an SVM predictor, which was trained based on only 14 experimentally proven Drosophila mirtrons, while Joshi *et al*.^27^ proposed an automated procedure for filtering introns for non-canonical miRNAs.

In this work we analyze over 900 miRNAs, propose a set of features to characterize pre-miRNA hairpins and explore the set of known mirtrons in a multidimensional feature space by applying PCA. We use selected features to train a group of machine learning-based predictors that are able to classify a pre-miRNA molecule as canonical or intron-derived. This project gives the basics for further development of a whole-genome mirtron predictor.

## Methods

In the study we used two datasets. First, the *miRBase set* (Supplementary Table S2) consisted of mirtrons and canonical miRNAs deposited in miRBase (Release 21, 06/14). To date Wen *et al*.^23^ provided the most comprehensive but also stringent mirtron/canonical miRNA annotation, therefore we used it in our study. From the database we extracted hairpin and mature miRNA sequences from both arms. We restricted the set to pre-miRNAs yielding functional mature miRNAs from both hairpin arms. The set contained 216 mirtrons and 707 canonical miRNAs. The second set we used, called *putative mirtrons set* (Supplementary Table S3) consisted of 201 novel mirtron loci annotated in study by Wen *et al*.^23^. Their sequences were gathered using UCSC browser - hairpin coordinates were made available in supplementary tables of Wen *et al*.^23^. Hairpin secondary structures and free energies for both sets were calculated using RNAfold (version 2.3.3) from ViennaRNA Package with default options.

### Training and test sets

Data from the *miRBase set* and the *putative mirtrons set* were used to construct the *training set* and the *test set*. In order to do so, 200 randomly chosen canonical miRNAs from *miRBase set* were merged together with the *putative mirtrons set*. These miRNAs formed the *test set*. The remaining miRNAs from *miRBase set* formed the *training set*. This approach resulted in total count of 723 (216 mirtrons/507 canonical miRNAs) in the *training set* and 401 miRNAs (201 mirtrons/200 canonical miRNAs) in the *test set*. The exploratory analysis and machine learning were performed on the *training set* while methodology validation on the *test set*.

### Feature definitions

**Figure 2:**
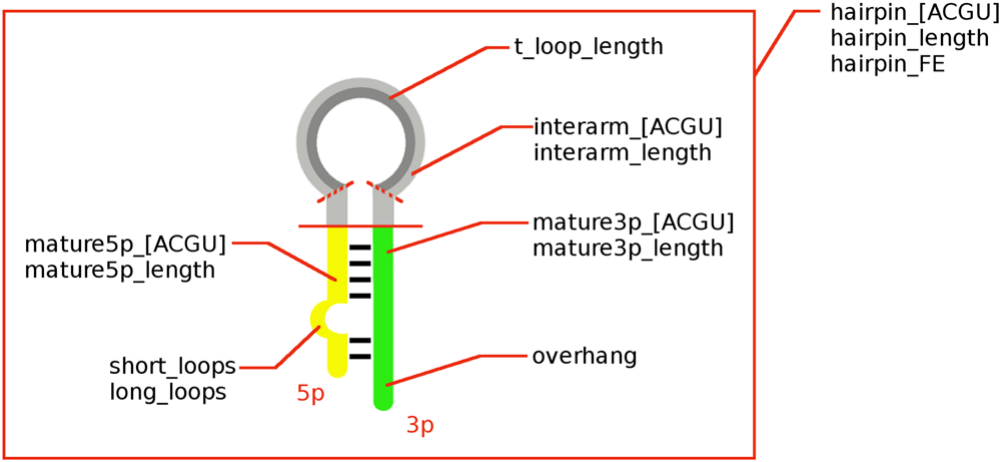
We proposed a set of 25 features to quantitatively characterize miRNA hairpins. We divided a model hairpin into three regions: the mature5p arm, mature3p arm and the interarm region. Each of the regions, as well as the whole hairpin, was characterized by its length and nucleotide content. Additionally, the hairpin is characterized by its free energy, number of short (<4 nt) and long loops (> = 4), the overhang and the length of the terminal loop. Hairpin_FE is the free energy calculated with RNAfold from the ViennaRNA Package. Overhang is the difference between number of unpaired nucleotides at the stem of the hairpin. Positive values indicate 5′ overhang while negative ones 3′ overhang.

We used 25 features for characterizing miRNA hairpins. The lengths of the hairpin and both arms of mature miRNAs were defined as the number of nucleotides within each region. Hairpin free energy was calculated using *RNAfold* from ViennaRNA Package which uses Minimum Free Energy algorithm (MFE)^41^. It was normalized by dividing it by hairpin length. Hairpin and mature miRNA nuleotide compositions were defined as percentages of each base occurring in a particular region. Interarm region was defined as the part of the hairpin between 5p and 3p arm where terminal loop can be found. We calculated its length and nucleotide composition. Overhang was calculated using mature sequences and predicted secondary structure. Positive values of the overhang refer to unpaired bases on the 5′ hairpin end, while negative values refer to unpaired bases on the 3′ end. We also calculated the numbers of small loops - sequence of less than 4 unpaired nt, large loops - sequence of more than 4 unpaired nt, and the length of the terminal loop. All features were calculated using an in-house R script (see “Data availability” section) and are shown in Fig. 2.

### Statistical comparison of feature distributions

We used Wilcoxon rank sum test for statistical comparison of distributions of calculated numerical features. We considered p-values below 0.01 as statistically significant.

### Data visualization

For data visualization we performed Principal Component Analysis (PCA). Linearly dependent features needed to be excluded from PCA calculations, therefore we arbitrarily decided to drop uracil compositions in all investigated hairpin regions, i.e. hairpin_U, mature5p_U, mature3p_U and interarm_U. The calculations were performed using the R *prcomp* function with prior data normalization. *ggplot2* package was used for plotting. The first two PCs explained 37,6%, while first three 46,8% of all variance.

### Classifier implementation and testing

We implemented six commonly used, methodologically diverse classifiers:

1. Logistic Regression calculated using *glm* function
2. Linear Discriminant Analysis using *lda* function from *MASS* package with default parameters
3. Support Vector Machine using *svm* function from *e1071* package with default radial kernel and default parameters
4. Naïve Bayes without smoothing using *naiveBayes* method from *e1071* package
5. Decision Tree without pruning using *tree* package
6. Random Forest using *RandomForest* package and default parameters (500 trees)

Classifier performance was measured using 5-fold cross validation.

For each of classifiers we calculated the following performance measures:

- Sensitivity

1 $$Sens=\frac{TP}{TP+FN}$$

- Specificity
  2 $$Spec=\frac{TN}{TN+FP}$$
- Area under curve (AUC) - Area under ROC curve
- F1-Score:
  3 $$F{1}_{score}=\frac{2\ast TP}{2\ast TP+FP+FN}$$
- Mathew’s Correlation Coefficient (MCC)

4 $$MCC=\frac{TP\,\ast \,TN-FP\,\ast \,FN}{\sqrt{(TP+FP)\,\ast \,(TP+FN)\,\ast \,(TN+FP)\,\ast \,(TN+FN)}}$$

### Feature importance

Analysis of feature importance was performed using three approaches: (i) single feature predictor accuracy, (ii) Stepwise Forward Selection (SFS) algorithm and (iii) Random Forest Importance calculated using Boruta package^42^. SFS was performed for the SVM classifier method with F1 score as the performance measure, while Boruta by definition uses Random Forest with Z-score importance returned.

### Methodology validation

The methodology was tested in a two-step analysis. Data points from the *test set* were projected onto the PCA biplot drawn for *training set*. To do this, *test set* points were first scaled with scaling parameters calculated for the first set and then projected onto PC1 vs PC2 plane using the transformation matrix calculated for *training set*.

In the second step we classified mirtrons and canonical miRNAs from the *test set* using classifiers trained on the *training set*.

### Data availability

Data tables containing studied dataset are available in CSV format in Supplementary Materials. The source code is freely available through GitHub (https://github.com/ror94/Mirtrons), distributed under the version 2 of the general public license (GPL v.2).

Since user-friendly and publicly accessible web-servers represent the future direction for developing practically more useful models^39,43–48^, we shall make efforts in our future work to provide a web-server for the method presented in this paper.

## Results

The aim of the study was to identify and explore the differences between canonical miRNAs and mirtrons using advanced computational tools. We also wanted to select a set of features that can possibly help determining whether particular miRNA sequences are derived from canonical or mirtron precursors. The study was based on two datasets: *miRBase set* and *putative mirtrons set* from which we constructed the *training* and *test* sets (for details see Methods).

We designed a set of 25 numerical features to characterize miRNA hairpins. These included features based on nucleotide content, free energy and structural motives. They are visualized in Fig. 2 (for detailed feature definitions see Methods).

**Figure 3:**
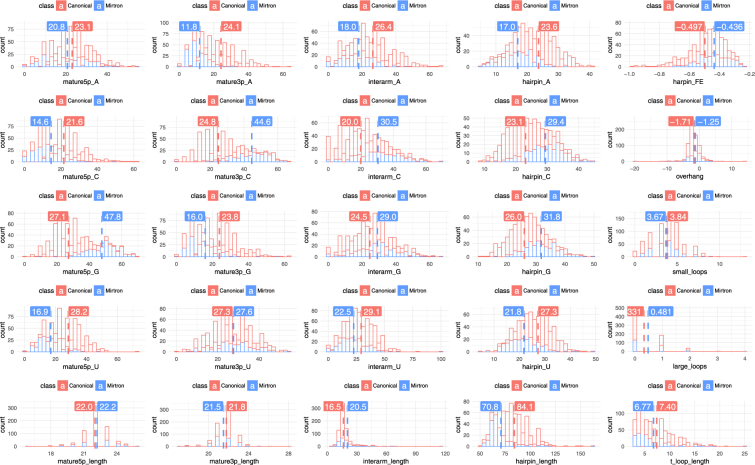
Histograms of all investigated features were produced with marked and labeled medians in R Studio. In columns there are features of mature 5p arm of mirtron, mature 3p arm, interarm region, precursor and miscellaneous features, in rows: A, C, G, U content and length. Features with greatest relative distance of means such as G content of 5p arm of mature miRNAs, C content od 3p arm and A content of whole hairpin structure are expected to carry most of information about a class of miRNA.

We first used a standard, non-parametric statistical test, Wilcoxon rank sum test to compare mirtrons and canonical miRNAs in the *training set*. The results indicated that the two groups differ significantly in terms of all but three features, i.e. uracil composition of 3p arm (mature3p_U), length of 5p arm of the mature miRNA (mature5p_length) and number of small loops (small_loops) (Table 1). Although average and median values of most features differ, their distributions strongly overlap, what makes it impossible to distinguish the two miRNA species using simple thresholds on any single feature (Fig. 3). Therefore multivariate analysis was used for further data exploration.

**Table 1:** The comparison of feature distributions in mirtron and canonical miRNAs was performed using Wilcoxon rank sum test implemented in R. Setting statistical significance to p-value less than 0.01 showed significance in all but three features: Uracyl composition of 3p arm of mature miRNA (mature3p_U), number of internal hairpin loops smaller than 4 nucleotide (small_loops) and the length of mature 5p arm (mature5p_length).

	name	Wilcoxon test	Mirtron median	Canonical median
1	hairpin_A	1.25 * 10^−35^	17.24	24.14
2	hairpin_C	1.64 * 10^−31^	29.69	22.58
3	hairpin_G	3.50 * 10^−30^	31.38	25.88
4	hairpin_length	4.23 * 10^−36^	67.00	83.00
5	hairpin_U	8.71 * 10^−26^	21.53	27.62
6	harpin_FE	4.56 * 10^−9^	−0.43	−0.48
7	interarm_A	8.90 * 10^−17^	18.14	25.00
8	interarm_C	7.62 * 10^−23^	28.57	18.75
9	interarm_G	1.28 * 10^−5^	28.57	25.00
10	interarm_length	3.50 * 10^−5^	17.00	16.00
11	interarm_U	5.08 * 10^−12^	21.43	28.57
12	large_loops	1.28 * 10^−3^	0.00	0.00
13	mature3p_A	1.11 * 10^−45^	10.00	22.73
14	mature3p_C	5.34 * 10^−66^	45.45	22.73
15	mature3p_G	5.69 * 10^−20^	14.29	22.73
16	mature3p_length	1.55 * 10^−5^	21.00	22.00
17	mature3p_U	9.32 * 10^−1^	27.27	27.27
18	mature5p_A	7.08 * 10^−3^	20.83	22.73
19	mature5p_C	2.62 * 10^−17^	13.64	21.74
20	mature5p_G	2.09 * 10^−70^	50.00	26.09
21	mature5p_length	8.76 * 10^−2^	22.00	22.00
22	mature5p_U	5.98 * 10^−39^	16.00	27.27
23	overhang	2.65 * 10^−10^	−1.00	−2.00
24	small_loops	1.19 * 10^−1^	4.00	4.00
25	t_loop_length	2.07 * 10^−3^	5.50	7.00

Medians of mirtron and canonical miRNAs were calculated to show the direction of differences.

**Figure 4:**
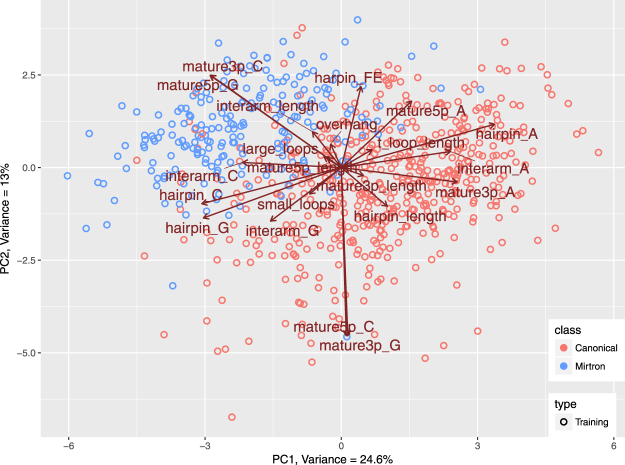
Principal Component Analysis performed on *training set* compressed over 37% of variance in first two components. It revealed separate grouping of mirtrons and canonical microRNAs and some hidden relations between variables and miRNA classes. In general, the features that contribute most to class distinction, are the ones whose vectors point in the direction of a particular group of molecules. Here those most important features are: cytosine in 3p arm (mature3p_C), guanine composition of 5p arm (mature5p_G), cytosine composition of interarm region (interarm_C), adenine composition of 3p arm (mature3p_A) and length of precursor (hairpin_length).

We explored datasets in the multidimensional space using PCA. PCA managed to compress the *training set*, so that 37,6% of all variance was captured in first two principal components (PCs) and 46,8% in first three PCs. In the two dimensional biplot we can observe that mirtrons and canonical miRNAs group separately. Feature vectors shown in Fig. 4 suggest that features with most contribution to separation are: mature5p_G and mature3p_C, which are higher in the mirtron group and hairpin_A, interarm_A, mature3p_A, mature3p_G and mature5p_C, which are higher in the group of canonical miRNAs. Apart from that, hairpin_length and mature3p_length seem to be important for the distinction, since they point clearly in the direction of canonical miRNAs.

In order to investigate the importance of designed features, we have built several standard, methodologically diverse classifiers: Logistic Regression (LR), Random Forest (RF), Linear Discriminant Analysis (LDA), Decision Tree (DT), Support Vector Machines (SVM), and Naive Bayes (NB). Almost all of them managed to classify properly both groups with sensitivity greater than 0.8 and specificity greater than 0.9 (Table 2). As number of samples in both groups were not equal, we used F1 score and Matthews Correlation Coefficient (MCC) as major parameters for assessing the classifier performances. Both metrics indicated that the two best classifiers are SVM and RF (Table 2). The results showed that combined features provide enough information to make the distinction between mirtrons and canonical miRNAs. We have also tested SVM classifier on the *test set* resulting in 186 True Positives and 15 False Negatives obtaining 0.93 sensitivity and 0.95 specificity (Table 3).

**Table 2:** Classifier performance comparison over all designed features.

	Method	Sensitivity	Specificity	AUC	F1	MCC
1	Support Vector Machines	0.926	0.945	0.935	0.901	0.859
2	Random Forest	0.870	0.957	0.914	0.883	0.836
3	Linear Discriminant Analysis	0.935	0.919	0.927	0.881	0.830
4	Logistic Regression	0.875	0.941	0.974	0.867	0.816
5	Decision Tree	0.861	0.943	0.902	0.863	0.808
6	Naive Bayes	0.875	0.894	0.884	0.824	0.746

Each classifier performance was evaluated using five metrics: Sensitivity, Specificity, Area Under Curve (AUC), F1-Score and MCC. Results are sorted by decreasing value of F1 and MCC.

**Table 3:** Confusion matrix of mirtron prediction using SVM model trained on 25 features.

		Reference	
		Mirtron	Canonical
Predicted	Mirtron	186	10
	Canonical	15	190

Prediction resulted in 0.925 sensitivity and 0.95 specificity.

We investigated the importance of particular features in three ways (i) using the performance of single feature predictors, (ii) using the SFS algorithm^49^ and (iii) using the feature selection algorithm in the Boruta package^42^ (for setup details see Methods). Boruta by its definition relies on Random Forest, while for single feature prediction and SFS we used our second best predictor - SVM.

Out of 21 features only top 11 single feature predictors acquired an MCC value greater than 0 and only top 7 had an AUC showing any meaningful predictive value (*AUC*¿0.6) (Table 4). Clearly single features are insufficient for distinguishing mirtrons and canonical miRNAs. This is consistent with observed distributions of feature values in Fig. 3. Among single feature predictors the best performing were based on: guanine content of 5p arm miRNA (sensitivity 0.699, specificity 0.921), cytosine content of 3p arm (sensitivity 0.653, specificity 0.925) and hairpin length (sensitivity 0.639 and specificity 0.864) (Table 4).

**Table 4:** Single feature predictors were built using Support Vector Machines (SVM) classifiers.

	Feature	Sensitivity	Specificity	AUC	F1	MCC
1	mature5p_G	0.699	0.921	0.810	0.742	0.646
2	mature3p_C	0.653	0.925	0.789	0.714	0.615
3	hairpin_length	0.639	0.864	0.752	0.650	0.509
4	mature3p_A	0.583	0.852	0.718	0.604	0.445
5	hairpin_A	0.362	0.937	0.649	0.476	0.380
6	hairpin_C	0.412	0.882	0.647	0.488	0.335
7	hairpin_G	0.366	0.907	0.637	0.453	0.324
8	interarm_C	0.213	0.943	0.578	0.312	0.244
9	interarm_length	0.129	0.966	0.548	0.206	0.179
10	harpin_FE	0.107	0.963	0.535	0.177	0.143
11	mature5p_length	0.107	0.935	0.521	0.161	0.067
12	mature3p_length	0.079	0.959	0.519	—	—
13	mature5p_A	0.000	1.000	0.500	—	—
14	mature5p_C	0.014	0.988	0.501	—	—
15	mature3p_G	0.033	0.992	0.512	—	—
16	interarm_A	0.009	0.996	0.503	—	—
17	interarm_G	0.019	0.986	0.502	—	—
18	overhang	0.139	0.925	0.532	—	—
19	small_loops	0.000	1.000	0.500	—	—
20	large_loops	0.000	1.000	0.500	—	—
21	t_loop_length	0.005	0.996	0.500	—	—

Each classifier performance was evaluated using five common metrics: Sensitivity, Specificity, Area Under Curve (AUC), F1-Score and Matthews correlation coefficient (MCC). Most of classifiers did not capture enough information to effectively classify mirtrons what resulted in very low sensitivity and high specificity. Only four classifiers were strong enough to provide a satisfying distinction - Guanine composition of 5p arm (mature5p_G), Cytosine composition of 3p arm (mature3p_C), length of precursor (hairpin_length) and Adenine composition of 3p arm (mature3p_A). These results are in line with statistical tests and PCA we performed on the dataset.

**Figure 5:**
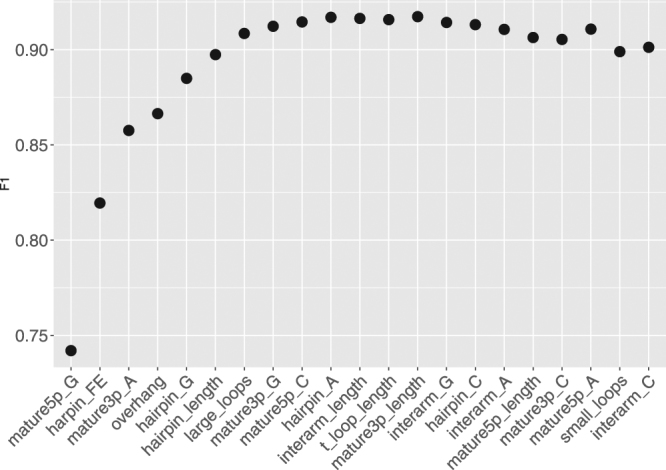
Feature selection using Stepwise Forward Selection procedure. In the procedure, features were sequentially added to the model in the way that maximized the F1 metric at each addition. The optimal subset contained first 13 features, for which the model acquired the best performance.

The top of the ranking delivered by Boruta was consistent with the ranking of single feature predictors showing that GC content and miRNA length related features were the most useful among others (Table 6). However there is an interesting difference in the rank of hairpin free energy (hairpin_FE), which is placed in the middle of single feature predictors ranking while being the 5-th most important feature according to the Boruta ranking. Such a discrepancy indicates that on its own, free energy is not discriminative with respect to canonical/non-canonical miRNA, however in conjunction with other features it significantly improves classification accuracy. It also shows that the information conveyed in the hairpin_FE feature is unique, since its randomization during Boruta importance estimation leads to a substantial drop of prediction accuracy. The SFS ranking similarly emphasizes the importance of hairpin_FE, which was ranked as the second most important feature. The top 5 of the SFS ranking also contains the overhang, which was in the middle of the ranking delivered by Boruta (Table 6). Figure 5 presents the changes upon addition of consecutive features in the SFS algorithm. The classification accuracy improves quickly during addition of the initial top 3 features. Then, it increases slightly upon addition of overhang and continues to improve afterwards. The optimal subset according to SFS algorithm contains 13 features. The F1 is approximately 0.92.

**Table 5:** Output from Stepwise Forward Selection algorithm.

	Feature	F1
1	mature5p_G	**0**.**742**
2	harpin_FE	**0**.**820**
3	mature3p_A	**0**.**858**
4	overhang	**0**.**866**
5	hairpin_G	**0**.**885**
6	hairpin_length	**0**.**897**
7	large_loops	**0**.**909**
8	mature3p_G	**0**.**912**
9	mature5p_C	**0**.**915**
10	hairpin_A	**0**.**917**
11	interarm_length	**0**.**916**
12	t_loop_length	**0**.**916**
13	mature3p_length	**0**.**917**
14	interarm_G	0.914
15	hairpin_C	0.913
16	interarm_A	0.911
17	mature5p_length	0.906
18	mature3p_C	0.905
19	mature5p_A	0.911
20	small_loops	0.899
21	interarm_C	0.901

F1 metric was the highest for the first 13 features, indicated in bold.

**Table 6:** Output from Boruta feature selection algorithm.

	Feature	Z-score
1	mature5p_G	**30**.**237**
2	hairpin_length	**24**.**944**
3	mature3p_C	**23**.**593**
4	mature3p_A	**22**.**983**
5	harpin_FE	**19**.**495**
6	hairpin_G	**14**.**645**
7	hairpin_A	**14**.**643**
8	mature3p_G	**14**.**441**
9	interarm_length	**13**.**555**
10	hairpin_C	**12**.**235**
11	mature5p_C	**10**.**992**
12	interarm_C	**10**.**225**
13	interarm_A	**9**.**177**
14	overhang	**8**.**863**
15	mature5p_A	**7**.**197**
16	interarm_G	**4**.**967**
17	small_loops	**4**.**488**
18	mature3p_length	3.043
19	mature5p_length	2.875
20	large_loops	2.520
21	shadowMax	2.315
22	t_loop_length	1.857
23	shadowMean	−0.023
24	shadowMin	−2.258

Scores significantly higher (*p* < 0.01) than scores of shadow attributes are indicated in bold.

**Figure 6:**
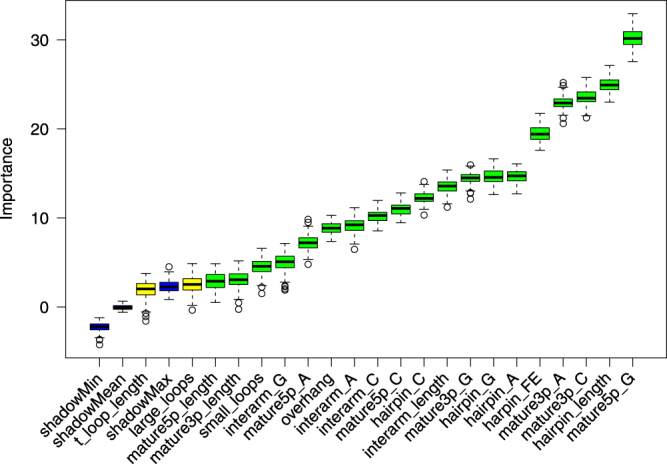
Blue boxplots depict minimal, average and maximum Z score of a shadow attribute. Yellow boxplots correspond to tentative attributes (t_loop_length, large_loops), whereas green ones represent confirmed features. TOP5 features stand out clearly from the rest: mature5_p_G, hairpin_length, mature3p_A, mature3p_C and hairpin_FE. Boruta measures the impact of randomizing a particular feature on the classifier performance, thus it may be used to asses the amount unique information that a feature encodes. The importance of the features mature3p_length and mature5p_length is comparable to shadow attributes, which serve as a baseline for feature usefulness.

Boruta also showed that the usefulness of some of designed features with respect to the classification task is doubtful, since their importance was comparable to randomly generated shadow features (Fig. 6). These features included large_loops and t_loop_length - marked as tentative, and mature3p_length, mature5p_length - having only marginally higher importance.

This analysis showed that a combination of several features is able to detect the specific pattern which allows distinguishing between the two classes of miRNA.

We retrained our classification models on the *training set* using the top 13 features from the SFS algorithm. This resulted in a meaningful performance improvement of all models (Table 7).

**Table 7:** Classifier performance comparison over top 13 features returned by Stepwise Forward Selection algorithm.

	Method	Sensitivity	Specificity	AUC	F1	MCC
1	Support Vector Machines	0.945	0.951	0.948	0.917	0.882
2	Random Forest	0.879	0.965	0.922	0.896	0.855
3	Linear Discriminant Analysis	0.940	0.925	0.932	0.888	0.840
4	Logistic Regression	0.884	0.941	0.976	0.874	0.823
5	Decision Tree	0.870	0.941	0.906	0.866	0.811
6	Naive Bayes	0.880	0.905	0.893	0.838	0.767

Each classifier performance was evaluated using five metrics: Sensitivity, Specificity, Area Under Curve (AUC), F1-Score and Matthews correlation coefficient (MCC). Results are sorted by decreasing value of F1 and MCC.

**Figure 7:**
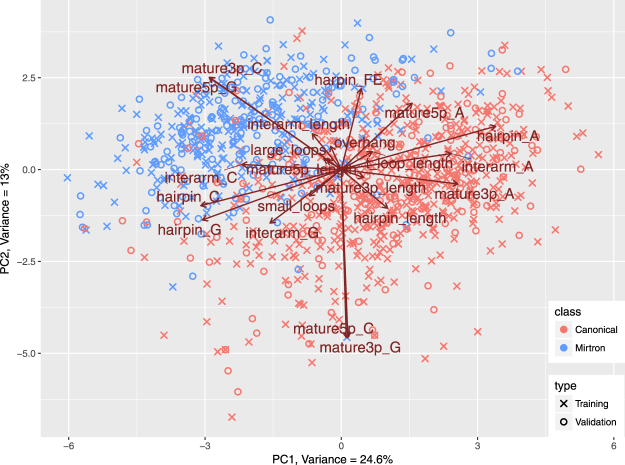
Datapoints representing proposed candidate mirtrons in study by Wen *et al*.^23^ were projected on the Principal Component space produced by PCA performed on *training set*. Generated biplot shows similarities between verified mirtrons and candidate in the space of chosen features as they strongly overlap on a plot.

Finally we validated the outcome of the study using the *test set* which consists of intron hairpins with a high potential of being mirtrons based on mappings of their genomic locations and numbers of reads^23^ and canonical miRNAs from miRBase that did not participate in preliminary data exploration. We used the transformation matrix derived from the *training set* PCA to calculate the PC coordinates of new samples. As shown in Fig. 7 the projected *test set* data (plotted as crosses) strongly overlap with samples from the *training set* (plotted as circles). This holds true in case of both, canonical miRNAs (red) and mirtrons (blue). This denotes in the investigated feature space the putative mirtrons are very similar to the confirmed mirtrons. In addition 184 out of 201 putative mirtrons (87%) and 189 out of 200 (95%) canonical miRNAs were correctly classified by our best classification model (Table 8).

**Table 8:** Confusion matrix of mirtron prediction using SVM model trained on 13 features.

		Reference	
		Mirtron	Canonical
Predicted	Mirtron	184	11
	Canonical	17	189

Prediction resulted in 0.915 sensitivity and 0.945 specificity.

## Discussion

The primary goal of the study was to explore the differences between canonical miRNAs and mirtrons. Both miRNA classes have the same biological role - post-transcriptional gene regulation, but mirtrons originate from a modified biogenesis pathway. To date various studies indicated high GC content in duplex regions and high free energy of mirtrons with respect to bulk introns^21,23,25^. These properties were confirmed in our analysis with respect to canonical miRNAs. Mature G and C content features were at the top of calculated feature importance rankings (Tables 4 and 6). Interestingly the features related to general hairpin nucleotide contents were not as informative. Although free energy in mirtrons was higher, the difference was not very pronounced. In the PCA plot the free energy vector pointed neither towards mirtrons nor canonical miRNAs (Fig. 4). A feature with such characteristics is not usually expected to contribute significantly to the classification accuracy. Still, the free energy was one of the most important features, ranked 5th by Boruta (Table 5) and 2nd by SFS (Table 6), meaning that its removal from the set of features would significantly lower the accuracy of distinction. Our study shows that nucleotide content features with addition of energy calculations detect an important mirtron specific pattern.

Investigation of length based features confirmed that the two classes of miRNA differ in terms of hairpin lengths. However, conversely to some studies^26,27^, in the analyzed *training set* the hairpins of canonical miRNAs were longer - median length of 83 nt in comparison to 67 nt in mirtrons (Table 1). The hairpin_length vector in the PCA plot points toward the canonical miRNAs indicating higher values in those molecules. In the study by Hung *et al*.^26^ the authors reported that bulges and long internal loops may be more prevalent in mirtrons and thus mirtron hairpins may be more similar to random hairpin sequences than canonical miRNAs. As a consequence penalization of unpaired regions when assessing mirtrons may not be appropriate. This suggestion was based on a much smaller mirtron dataset i.e. only 14 D. melanogaster mirtrons. Our results do not support this fact. The features that quantify loop presence and lengths were not significant in statistical tests of differences (Table 1) also their importance in classification was low (Tables 5 and 6), which implies that mirtrons and canonical miRNAs share similar characteristics in terms of internal loops and bulges. Another important miRNA feature is the overhang. The typical 0:2 overhang in canonical miRNAs is a result of the Drosha cleavage^4^. Mirtrons bypass this part of miRNA genesis pathway. Therefore one might expect that there might be a difference in terms of overhang length. For instance mirtrons that are derived directly from splicing were reported to have a 1:1 nucleotide overhang^20^. In the explored *training set* the overhang proved to be beneficial for the classification (ranked 4th in the SFS ranking). However the fact that it was ranked in the middle of Boruta ranking denotes that the information it carries may also be encoded in some other features. Such redundancy would explain its lower impact on classification accuracy as measured by Boruta.

High classification accuracy produced by all tested machine learning methods (Table 2) shows that mirtrons form a distinct group of molecules that can be confidently distinguished from canonical miRNAs based on the proposed features. Moreover, we showed that it is possible to reduce the set of features to a subset of 13 features, with special emphasis on the most pronounced properties differing the analyzed miRNA types, i.e. the G content in the mature 5p arm, the hairpin length, the A and C content in mature 3p arm and hairpin free energy.

The PCA projection of *test set* showed that putative mirtrons group together with miRBase mirtrons. Moreover, classification of putative mirtrons resulted in 87% of samples classified as mirtrons. Although these results cannot be perceived as a strict test of accuracy, they show that annotation based on hairpin sequence features correlates well with the outcome of genetic location annotation. This supports the validity of the proposed approach of hairpin characterization and suggests that it may be possible to improve prediction of new mirtrons using computational tools.

## Conclusion

In this work, we proposed a set of quantitative features for characterizing miRNA hairpins. We used PCA, machine learning classifiers and feature selection algorithms to identify and explore the differences between mirtrons and canonical miRNAs. The most important differences were related to nucleotide content in the duplex region combined with hairpin free energy. Clearly, the 5p arm mature regions of mirtrons were richer in Guanine and simultaneously, their 3p arms were richer in Cytosine. On the other hand the mature 3p arms of canonical miRNAs were shown to be richer in Adenine. In addition mirtrons were characterized by higher free energy levels and shorter hairpin lengths. Although our study is consistent with the outcomes of several experimental works on mirtron/canonical miRNA differences, we cannot confirm that the two miRNA classes differ in terms of bulges and internal loops.

Our results show that sequence-based miRNA classification is consistent with genomic location-based annotation. This work will be used as a starting point for further *in silico* mirtron prediction.

## Electronic supplementary material

Supplementary Table S1

Supplementary Table S2

Supplementary Table S3
